# Case report: Dissociative neurological symptom disorder with gait disturbance: taking after the father?

**DOI:** 10.3389/fpsyt.2024.1359510

**Published:** 2024-05-24

**Authors:** Wenqi Geng, Yinan Jiang, Jing Wei

**Affiliations:** Department of Psychological Medicine, Peking Union Medical College Hospital, Chinese Academy of Medical Sciences and Peking Union Medical College, Dongcheng, Beijing, China

**Keywords:** dissociative neurological symptom disorder, conversion disorder, functional neurological disorder, gait, referral and consultation, suggestion therapy

## Abstract

Dissociative neurological symptoms disorder (DNSD), or conversion disorder, frequently manifests with unexplained neurological symptoms, necessitating referral to psychiatry following preliminary diagnosis in neurology. We present a case of an adolescent female patient with gait disturbance as the predominant clinical presentation, and delve into the diagnosis and interdisciplinary intervention process. Given neuroimaging deviations detected and familial similar presentations, the organic etiology was confirmed. However, the aberrant gait remained unexplained ultimately prompting psychiatric consultation resulting in the diagnosis of DNSD. Interventions consisting of health education, suggestive therapy, and physiotherapy notably improved gait disturbance. However, at follow-up, the patient presented with a depressive episode. It was deduced that undiagnosed psychosocial factors, notably familial dynamics, likely contributed to this decline. Eventually, transformed relation patterns among family members as well as antidepressant treatment were instrumental in attaining symptom remission.

## 1 Introduction

Dissociative neurological symptom disorder (DNSD), or functional neurological symptom disorder, is characterized by the presentation of motor, sensory, or cognitive symptoms that imply an involuntary discontinuity in the normal integration of motor, sensory, or cognitive functions, lasting at least several hours (1, 2). DNSD include different manifestations such as paresthesia, motor abnormality and cognitive symptoms. DNSD with gait disturbance is characterized by symptoms involving the individual’s ability or manner of walking, including ataxia and the inability to stand unaided. DNSD typically initiates during adolescence and early adulthood, may arise subsequent to traumatic or adverse life incidences, and prior neurological disease is also a risk factor (1, 3, 4). In adolescent patients with DSND, gait disturbance and non-epileptic episodes are predominant and are frequently associated with mood disorders, anxiety, or fear-related disorders (1, 5–10). In this manuscript, we present and discuss the diagnosis and management of an adolescent patient with organic neurological disease who concurrently manifested with dissociative gait abnormalities. To our knowledge, this is the first case report of an adolescent Chinese patient with DNSD with gait disturbance.

## 2 Case presentation

A 15-year-old female patient presented with intermittent gait disturbance of five months. She initially exhibited fevers, diarrhea, dizziness, and nausea during the second half of grade nine, seven months earlier. A diagnosis of infectious fever was made. The etiology remained unidentified, yet, the symptoms notably declined post-treatment; she returned to school following a brief leave. Two months later, the patient reported general discomfort and fatigue. She developed sudden unsteady gait which affected walking, despite being able to stand still. The diagnosis was determined via an MRI revealing abnormal neuroradiological signals of C1–7, suggestive of myelitis. Management incorporated oxacillin sodium 3g twice daily for one week, followed by dexamethasone 5mg once daily for another week. Although the pain and fatigue decrease, the abnormal gait persisted. Given the lack of a precise diagnosis from the neurologist, specialized therapy was deferred, yet gait exercises were encouraged, leading to a stable period free of gait disturbance lasting 3 months, including the pre-high school summer break. Two months later, post-high school enrollment, the patient experienced a recurrence of abnormal gait. Given this, she was admitted to our hospital’s neurological department for comprehensive assessment and management. The neurological examination revealed abnormal gait, characterized by restricted base, unstable ambulation, balance impairment, and difficulty with heel and toe maneuvers. Notably, gait deficits fluctuated in severity. Additional neurologic manifestations included reduced sensation at the left palm’s distal region, right wrist, and both ankles. Neuropathic reflexes, meningeal irritation indicators, cranial nerves, and autonomic nervous system examinations exhibited no significant anomalies. During mental status examination, the patient was passive in communication, and was worried about her gait disturbance. No history of depression or anxiety was reported. Regarding her likely causes for symptoms, the patient speculated on “nerve issues”. Stressors such as academic pressures were initially deemed insignificant.

**Figure 1:**
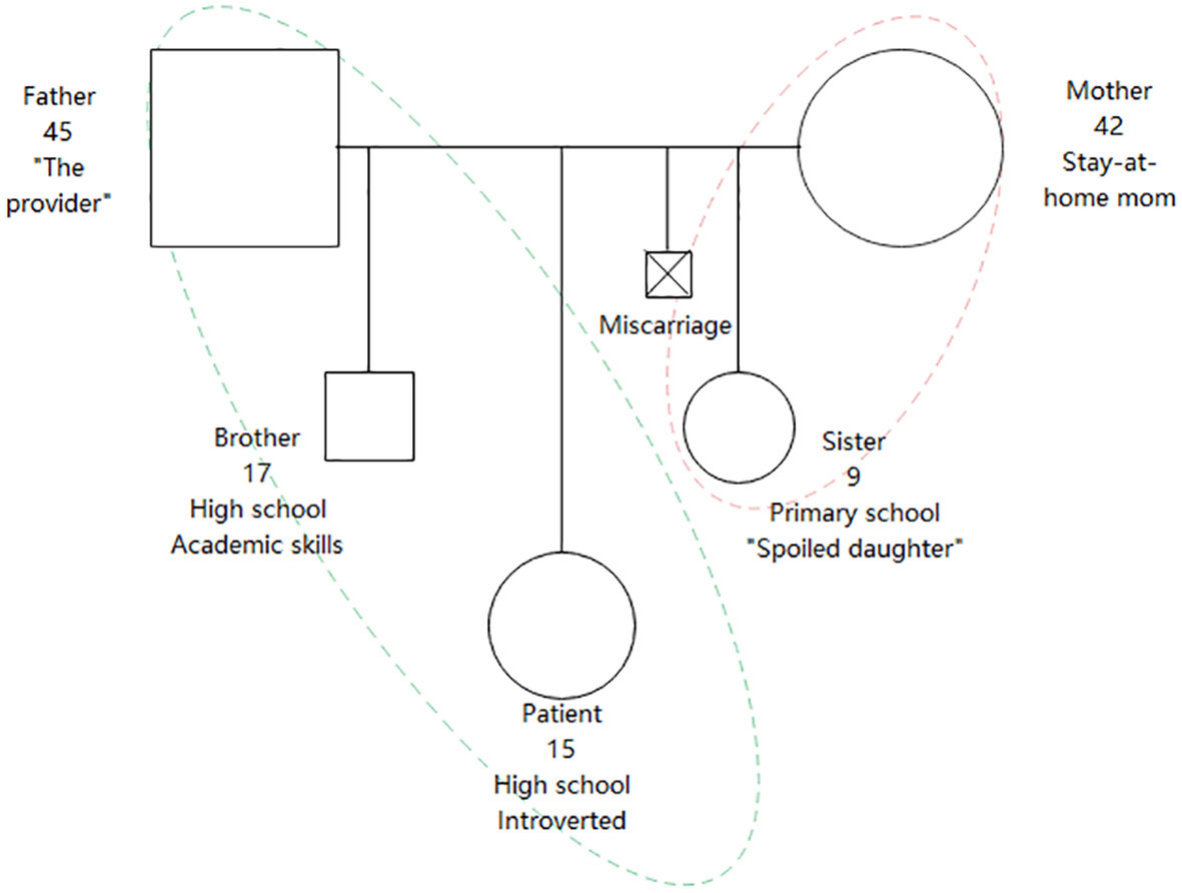
Core family genogram of the patient.

Previously in good health and developing normally from infancy, the patient resided in rural area with no known exposure to livestock or raw milk products. Her father presented with fever and limb paresthesia two years prior, subsequently diagnosed with brucellosis, and made a full recovery post-treatment. The patient’s elder brother suffered from similar symptoms, including fever, diarrhea, pain and numbness in the lower limbs, and fatigue, resolving spontaneously within a month without elucidation of the underlying cause. No substance use was reported. The patient has a younger sister in addition to their brother. The patient attended a boarding school. Her older brother also attended boarding school, excelling academically, fostering parental expectations for similar performance from the patient. Her younger sister, considered as “the spoiled daughter”, attended primary school and was particularly attached to their mother. Normally, the father worked in another city, returning home infrequently to visit his family. The mother diligently handled household responsibilities and agricultural tasks simultaneously. The patient maintained a strained relationship with her mother yet a strong bond with her father. There was minimal communication among family members, occasionally the patient would discuss school life with her brother. Upon onset of the patient’s illness, the father ceased working and temporarily reunited with the family. Upon seeing initial improvement in the patient’s condition, he left for work again. The patient was introverted and reserved. She had excellent academic performance prior to illness and hospitalization. Nevertheless, her high school entrance examination result was unsatisfactory, potentially due to inconsistent school presence. The patient attended a subpar high school; she forged new friendships and achieved improved academic performance. Her mother experienced depressive symptoms after a miscarriage, without seeking professional help. No other family members had a history of mental disorders. Aside from the brucellosis of the father, no notable physical disease familial history existed. The genogram of the patient is shown in **Figure 1**.

Laboratory blood tests, including complete hemogram, comprehensive metabolic panel, infection markers, and autoimmune markers, yielded results within the normal range. Cerebrospinal fluid tests turned out negative while brucella agglutination tests were also negative. Electromyography indicated no neurogenic or myogenic injury. Cervical spine MRI noted suspected abnormal enhancement at C3–4 level. During psychological evaluation, the patient had elevated scores in hysteria, depression, and dependency on the Minnesota Multiphasic Personality Inventory (MMPI). The patient scored 89 in Toronto Alexithymia Scale, which indicated alexithymia. Defensive mechanisms showed heightened somatization, dissociation, and avoidance trends. Stress indicators on the Adolescent Life-Change Events Scale were identified in interpersonal relationships, health adjustments, and academic stress, although none proved significant.

The attending neurologist postulated that the patient’s prior history of fever, diarrhea, pain and numbness in lower limbs all fell within the realm of an infectious process. Upon admittance, the patient was approximately in the phase of recovery with no inflammation evident in the serum or cerebrospinal fluid. There was a discrepancy between the gait abnormality and disease progression as well as inconsistencies in physical examination findings. Consequently, it was concluded that gait symptoms unlikely stemmed from an infection, but rather, functional symptoms were plausible. Following a psychiatric evaluation, the patient’s aberrant gait performance matched the diagnostic criteria for DNSD with gait disturbance in ICD-11 (1). Following a joint assessment by the psychiatrist and neurologist, the treatment plan was explained to the patient and family regarding dissociative symptoms health education. Psychiatrists primarily undertook psychological education, elucidated the complexities of patient conditions, acknowledged symptom distress, and elucidated that a diagnosis of functional symptoms does not signify symptom simulation and can be ameliorated through therapy. Notably, emphasis was placed on avoiding persistent impairment in social functioning due to symptoms, and post-suggestion therapy led by a neurologist, the patient was encouraged to persistently engage in rehabilitation therapy. The neurologist’s medical team then assumed the role of the “medical authority”, prescribing drug suggestion therapy for the patient. A slow intravenous infusion of calcium gluconate and saline ensued, after which the patient was encouraged to practice walking. The patient exhibited immediate improvement in gait, able to walk independently despite a sluggish pace. Subsequently, prior to discharge, the rehabilitation department directed daily lower limb strengthening for three days, leading to improved gait normalization. At discharge, the patient had achieved normal gait.

Post-discharge at a 3-week follow-up, the patient exhibited pervasive depressed mood lasting over 2 weeks, coupled with intermittent dissociative amnesia and self-injury behavior, including self-bruising, reported by the parents. The patient could not recall any of these incidents. The psychological evaluation revealed a Hamilton Depression Rating Scale-17 score of 19. The psychiatrist reassessed the patient’s mental condition and psychosocial factors, assumed that unaltered factors attributing to dissociative symptoms exacerbated the psychiatric state again. Given the constraints of time, the patient and her family consensually agreed to undergo one session of family psychotherapy, which included the patient and her parents. In this therapeutic session, the therapist maintained empathic presence and active listening, initially acknowledging the distress the patient experienced with her illness, as well as the family’s distress. Subsequently, the patient and her parents vocalized their respective attributions for the patient’s disease and their perspectives towards the factors impacting the condition. Since all three members believed that the family relationship and academic pressure may have influenced the patient’s condition, the therapist facilitated a collective discussion within the family about potential strategies to ameliorate these two issues, and reached consensus on the final plan. The ultimate decision of the family was for the father to assume more responsibility for caring for the family, while encouraging the patient to persist in attending school. The family also adopted the therapist’s recommendation to incorporate the practice of articulating emotional feelings into their post-therapy home assignments. Alongside, antidepressant therapy was prescribed at a dose of sertraline 100mg daily. The patient was further directed to conduct relaxation exercises thrice daily via breathing and meditation techniques. Post-discharge at five weeks, the patient’s depression showed partial improvement and she resumed academic activities.

**Figure 2:**
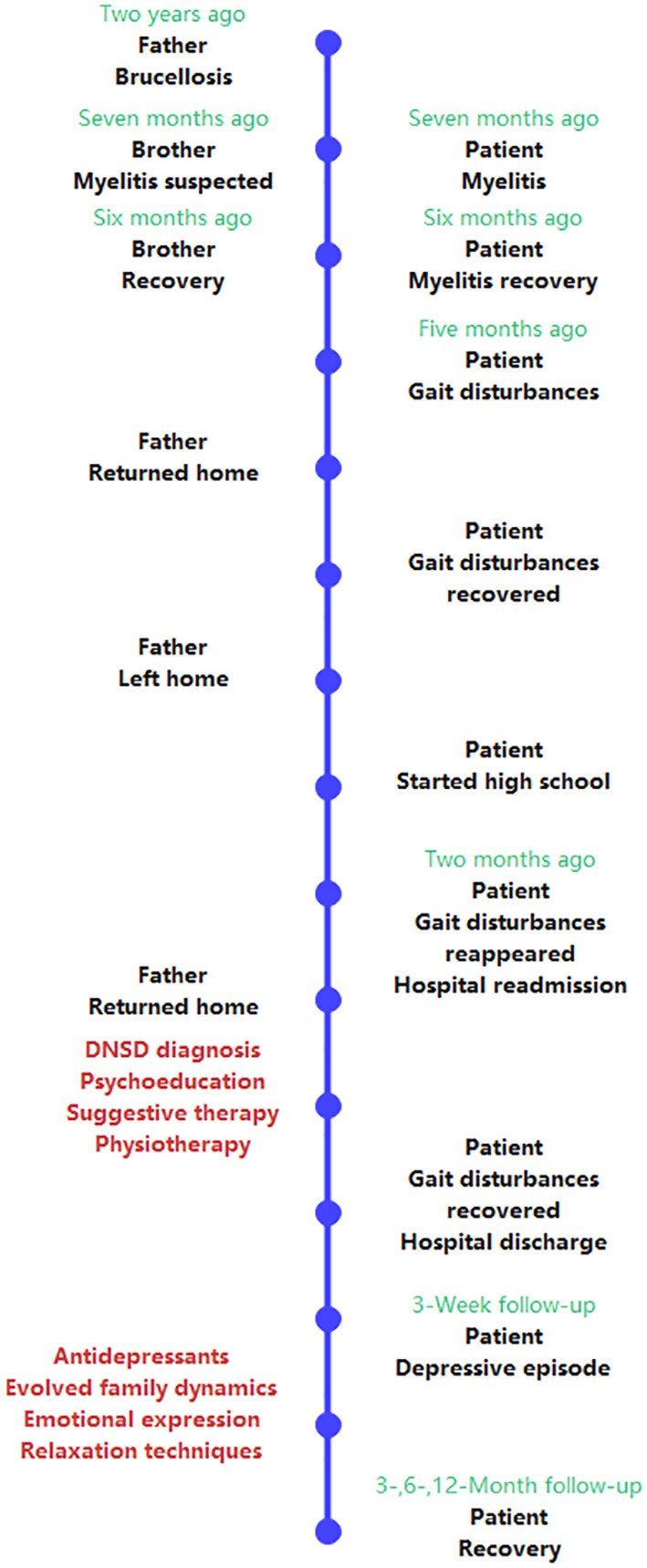
Timeline of the case. DNSD, Dissociative neurological symptom disorder.

Follow-ups conducted two and three months later confirmed clinical remission from previous depressive episode and alleviation of dissociative symptoms. During the six-month follow-up, the patient mentioned her participation in the relay race of a sports meet on behalf of her class and had developed stable friendships. Although she was still stressed about academic performance, her mood remained stable. Her father had adjusted his work arrangements, ceased traveling to other cities and instead worked locally, allowing the patient to reunite with her parents weekly as she returned home from boarding school, reducing the stress on the mother’s care for the child and enabling her to seek part-time employment. At the one-year follow-up, the patient’s physical and mental state were satisfactory, and she did not actively discuss her family but focused more on her life at school and relationships with friends. She considered herself a trusted friend by her peers and began participating in some group activities. When inquired, she described her parents now engaging in more arguments than before, but these disputes “were trivial matters, insignificant,” and she believed that her parents’ relationship was better than before. Her brother had enrolled in a university outside the city, returning home for winter and summer breaks and occasionally speaking with her. She felt that her relationship with her sister was closer than before, as her sister preferred to cling to her rather than their mother. She did not perceive a closer bond with her mother. She found her father’s advice to be somewhat excessive, causing her some annoyance, but still considered their relationship to be positive. The patient’s diagnostic and treatment path is depicted in **Figure 2**.

## 3 Discussion

The diagnosis of DNSD is typically confirmed by a neurologist in collaboration with a psychiatrist. In this case report, an adolescent female patient exhibited both organic and functional neurological symptoms. Pain and numbness in lower limbs and fatigue were manifestations of the myelopathy, while abnormal gait is the sole unexplained symptom, which is also the patient’s most distressful complaint. There are two facts suggesting that the patient’s gait disturbance may be functional symptoms. One is the instability of the patient’s physical signs and their discrepancy from objective assessments. The other is that the symptoms can be ameliorated by suggestion therapy and general rehabilitation training, which do not usually occur in gait abnormalities induced by neurological disorders. In reviewing the patient’s medical history, the patient’s functional gait symptoms might have been potentially detected earlier, especially given the patient’s return to normal gait without specialized treatment. Regrettably, this clinical phenomenon did not receive due consideration by the neurologists at that time. It was only when the gait symptoms reappeared and the patient was transferred to a higher-level hospital that we were invited as psychiatrists for consultation, and the impact of the patient’s psychosocial factors on her condition beyond the physical level was first noted. Neurologists are not deficient in theoretical knowledge and experience of diagnosing functional symptoms, but they often struggle with recognition and interventions targeting patients’ psychosocial factors. Hence, interdisciplinary collaboration is paramount for the optimal treatment of DNSD.

Adolescence is the stage of life with elevated occurrence of DNSD, often coexisting with mood disorder (1, 5, 11). Simultaneously, individuals with neurological diseases may exhibit functional symptoms, such as epileptic patients experiencing non-epileptic seizures or panic attacks. The underlying psychopathological mechanisms are notably complex (12, 13). Adolescent patients encounter multiple stressors, including academic, social, and familial (11, 12). Past studies have indicated that relationships with one’s family are often a contributing factor in adolescents with DNSD (14, 15). Children and adolescents are particularly vulnerable because of an immature personality and increased susceptibility to adverse situations, especially involving family issues (12, 15). Concurrently, because dissociative symptoms may yield primary and secondary benefits, if the patient do not receive prompt and adequate intervention, these symptoms may develop into chronic condition, which significantly affects one’s function (16). Studies suggest that iatrogenic interventions may not match the efficacy of significant life changes (17); conversely, enduring adverse life changes due to extended illness may denote unfavorable clinical outcomes (13). In the case report of an adolescent male patient with functional syncope comorbid with cardiac disease, the authors also noted that timely diagnosis and psychiatric intervention may improve the patient’s clinical course, mitigate psychological distress, enhance psychosocial functioning, and reduce school absenteeism (13). In our clinical case, the patient’s myelitis may have been precipitated by his father’s infectious disease, while unchanged familial dynamics might be implicated in the fluctuations of the patient’s dissociative manifestations. Following abnormal gait symptoms, the patient was able to evade academic stress and acquire more parental attention, thereby fulfilling her unconscious wishes and relieving the symptoms. Following the second hospital admission and amelioration of gait abnormalities, the patient encounters multiple stressors again, but this time both the patient and family have comprehended the factors influencing the dissociative symptoms. As a consequence, more primitive conversion symptoms did not reappear, instead mood episodes were presented. In another view, the patient no longer articulates pain physically, but emotionally. This suggests improvement in patient’s alexithymia post-treatment, echoing prior research findings (18).

Unchanged family dynamics have impeded the patient’s comprehensive recovery. Through a single session family therapy at follow-up, the patient and her parents jointly opted to alter the relation patterns within the family. When the father subsequently invested more time in the family, the family dynamics began to evolve. The responsibilities previously shouldered by the mother were partially transferred to the father, while the father-daughter bond’s supportive function for the patient was enhanced. The mother acquired more leisure time outside the family, the relationship between the mother and the younger daughter became appropriately distant, and the sibling bond between the patient and her sister deepened, contributing to the patient’s acquisition of more emotional closeness, potentially replacing the mother-daughter relationship. In recent years, some studies have indicated that single-session therapy effectively treated mental disorders such as anxiety, depression, and eating disorders and physical diseases (19–22). This approach is particularly beneficial for patients who may not be able to receive comprehensive psychotherapy due to limitations in their circumstances (19, 20). We propose that single therapy or ultra-short-term psychotherapy could significantly contribute to the liaison-consultation process especially in general hospitals, as demonstrated by this case study.

In this case, physical rehabilitation yielded a beneficial impact on ameliorating dissociative symptoms; prior studies have also observed similar outcomes (23–25). Complex treatment including behavioral training, family therapy, medication in case of concomitant disorders, and rehabilitation, brings the best therapeutic effects (26, 27). One previous research has discovered that psychiatrists are poorly informed regarding advancement in research pertaining to DNSD, such as insufficient comprehension of biological mechanisms, and inadequate focus on emerging interventions like biofeedback and physiotherapy (28). We propose that physical rehabilitation therapy, as a physiotherapy, can potentially be widely employed in patients with dissociative movement disorders in the future.

The diagnostic evaluation and management of DNSD should include multidisciplinary teams encompassing neurology, psychiatry, and rehabilitation, and highlight the comprehensive psychosocial elements of the patient, particularly the familial elements in adolescent patients. Health education, suggestion therapy, relaxation techniques, physical rehabilitation and other comprehensive interventions contribute to optimal clinical outcomes.

## Data availability statement

The original contributions presented in the study are included in the article/supplementary material, further inquiries can be directed to the corresponding author/s.

## Ethics statement

The studies involving humans were approved by Ethics Committee of Peking Union Medical College Hospital. The studies were conducted in accordance with the local legislation and institutional requirements. Written informed consent was obtained from the individual(s) for the publication of any potentially identifiable images or data included in this article.

## Author contributions

WG: Conceptualization, Data curation, Methodology, Writing – original draft, Writing – review & editing. YJ: Conceptualization, Investigation, Methodology, Supervision, Writing – review & editing. JW: Funding acquisition, Supervision, Writing – review & editing.
